# Isolated Hemichorea-Hemiballismus as the Presenting Manifestation of Thalamic Intracerebral Hemorrhage: A Long-Term Treatment Course With Tetrabenazine and Aripiprazole

**DOI:** 10.7759/cureus.115766

**Published:** 2026-09-04

**Authors:** Saraa Zarin, Adrian Parry-Jones

**Affiliations:** 1 Neurology, Salford Royal NHS Foundation Trust, Salford, GBR; 2 Faculty of Biology, Medicine and Health, University of Manchester, Manchester, GBR; 3 Stroke Medicine, Geoffrey Jefferson Brain Research Centre, Salford Royal NHS Foundation Trust, Salford, GBR

**Keywords:** aripiprazole, hemichorea-hemiballismus, intracerebral hemorrhage, post-stroke movement disorder, stroke, tetrabenazine, thalamic hemorrhage

## Abstract

Post-stroke movement disorders are uncommon, and hemichorea-hemiballismus is most often associated with lesions involving the basal ganglia or subthalamic region. Thalamic lesions are a recognized but less frequent cause. We report a 78-year-old right-handed woman who developed sudden involuntary jumping and dancing movements of the right foot, which later spread to the right arm. She had no weakness, sensory loss, dysarthria, visual symptoms, headache, or loss of consciousness. Examination showed continuous right-sided hemichorea-hemiballismus with preserved strength and sensation. Non-contrast computed tomography demonstrated a focal acute left thalamic intracerebral haemorrhage, with no additional haemorrhage, mass effect, midline shift, or acute infarction identified on the initial scan.

Four-factor prothrombin complex concentrate was administered for reversal of edoxaban-associated anticoagulation, and anticoagulation was initially withheld. Tetrabenazine was started and gradually increased to a maximum of 25 mg three times daily, with substantial improvement in the involuntary movements and participation in rehabilitation. Attempts to reduce the dose were followed by worsening of the movements. At higher doses, however, she developed significant irritability, low mood, and emotional lability. Tetrabenazine was therefore gradually withdrawn, and aripiprazole was started at 2.5 mg daily and increased to 7.5 mg daily. At subsequent follow-up, the hemichorea remained markedly improved, and her mood improved after tetrabenazine was discontinued. Mild choreiform movements persisted beyond 12 months, and aripiprazole was later increased to 10 mg daily.

This case shows that a focal thalamic haemorrhage can present with isolated hemichorea-hemiballismus and that symptoms may remain clinically relevant for more than a year. It also highlights the need to balance symptomatic benefit against neuropsychiatric adverse effects when using tetrabenazine and to consider alternative treatment when symptoms persist.

## Introduction

Movement disorders are uncommon complications of cerebrovascular disease. Post-stroke movement disorders include chorea, ballism, dystonia, tremor, myoclonus, and parkinsonism, with involuntary movements occurring after approximately 1-4% of strokes in published series and reviews. Hemichorea-hemiballismus is among the most frequently reported hyperkinetic movement disorders after stroke and may develop either acutely or after a delay [1-4].

Hemiballismus was classically associated with lesions of the contralateral subthalamic nucleus. However, clinicoradiological studies have shown that similar movements can arise from lesions involving other parts of the basal ganglia-thalamocortical motor system, including the striatum, globus pallidus, thalamus, and cortex [5,6]. Lesion-network studies further suggest that hemichorea-hemiballismus may result from disruption of a wider motor network rather than injury to a single anatomical structure [7]. A systematic review of movement disorders following thalamic stroke found that acute presentations are well recognized and that the posterolateral thalamus is commonly involved [8].

Thalamic hemorrhage presenting predominantly with hemichorea-hemiballismus remains uncommon [8,9]. A recent case report described hemiballismus as the sole manifestation of a thalamic hemorrhagic stroke [9]. The present case adds to the limited literature by describing a prolonged treatment course extending beyond 12 months, including clinically observed, dose-related improvement with tetrabenazine, recurrence of symptoms after dose reduction, treatment-limiting neuropsychiatric adverse effects, and subsequent treatment with aripiprazole.

## Case presentation

A 78-year-old right-handed woman presented with sudden-onset involuntary movements affecting the right side of her body. Her medical history included hypertension, atrial flutter treated with edoxaban, type 2 diabetes mellitus, chronic kidney disease stage 3 [10], hypercholesterolaemia, asthma, glaucoma, and age-related macular degeneration. Before the event, she lived at home with her husband and was independently mobile, with a premorbid modified Rankin Scale score of 2 [11]. No pre-existing psychiatric diagnosis was documented in the available medical records.

Two days before presentation, she had sustained a minor fall with a head injury but was able to get up and returned to her neurological baseline. The following day, she felt somewhat unsteady while mobilizing. Later that afternoon, her husband noticed involuntary jumping and dancing movements of the right foot, which subsequently spread to the right upper limb. She reported no new headache, visual disturbance, change in speech or swallowing, limb weakness, or altered sensation. There had been no recent medication changes, and there was no personal or relevant family history of chorea or another movement disorder.

On initial assessment, her blood pressure was 166/76 mmHg, capillary blood glucose was 6.5 mmol/L (local inpatient target range for people with diabetes: 4-12 mmol/L), and Glasgow Coma Scale score was 15/15. Neurological examination demonstrated continuous irregular involuntary movements of the right upper and lower limbs. Specialist neurological review described large-amplitude, non-rhythmic hyperkinetic movements with hemiballistic features. Tone was normal, power was preserved at Medical Research Council grade 5/5 throughout, and sensation was intact. Cranial nerve examination showed normal eye movements and visual fields, no facial weakness, and normal speech. There was no neglect or inattention. Coordination testing was limited by the marked right-sided involuntary movements, and no separate cerebellar syndrome was documented. Reflexes and a formal gait assessment were not recorded in the contemporaneous initial neurological examination. In the absence of weakness, sensory loss, cranial nerve deficit, dysarthria, neglect, or inattention, the presentation was considered consistent with isolated right-sided hemichorea-hemiballismus.

Non-contrast computed tomography of the brain demonstrated a focal acute haemorrhage within the left thalamus, measuring approximately 13 mm, with no additional parenchymal haemorrhage, extra-axial collection, mass effect, midline shift, acute or recent infarction, or skull fracture (Figure 1).

**Figure 1:**
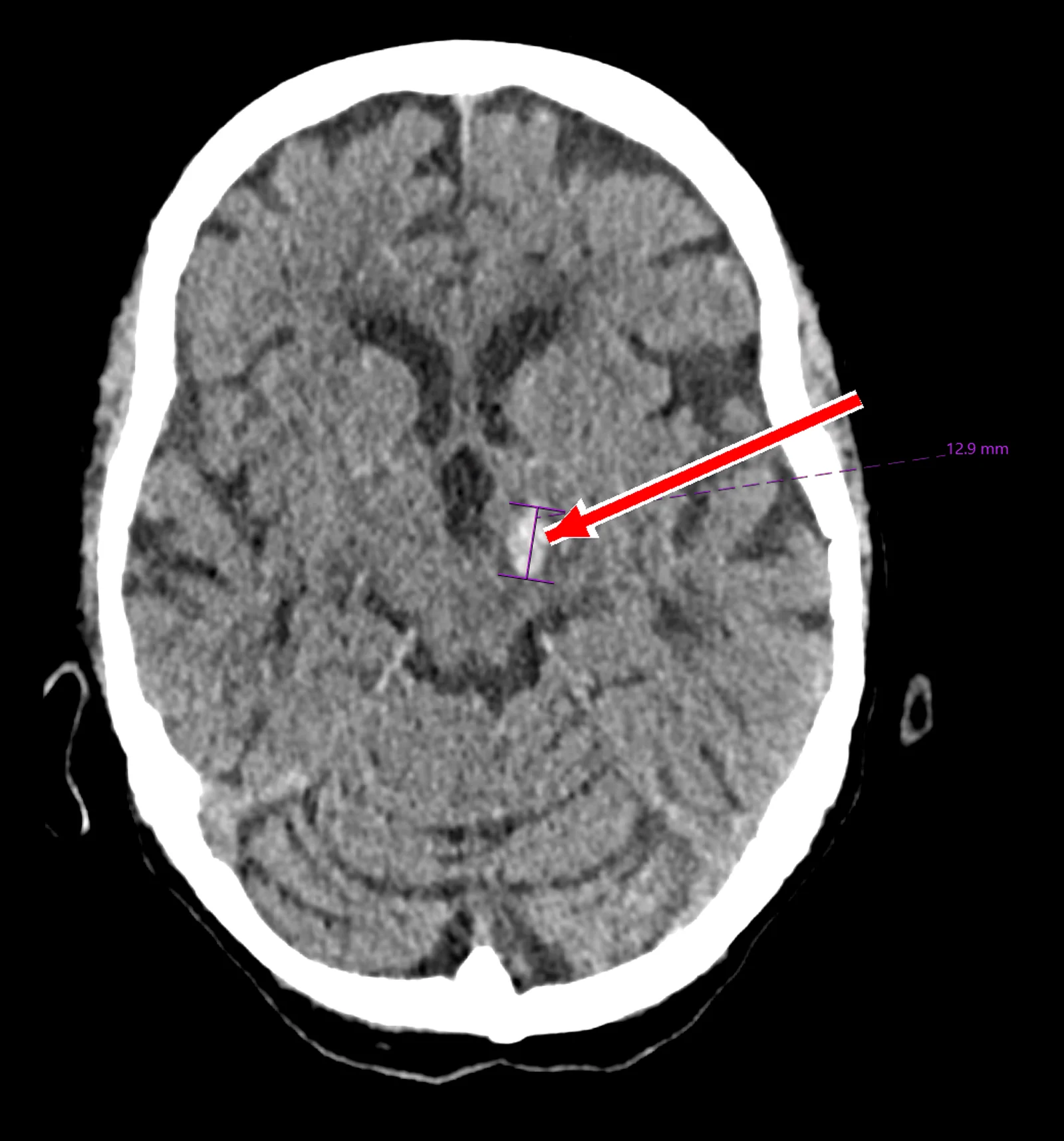
Acute left thalamic intracerebral hemorrhage on CT. Non-contrast axial CT at presentation demonstrating a focal acute hemorrhage centered within the left thalamus, measuring approximately 13 mm in maximal dimension. The arrow indicates the left thalamic hemorrhage.

As she was taking edoxaban for atrial flutter, the case was discussed with the hematology team, and four-factor prothrombin complex concentrate (Octaplex) was administered for reversal of anticoagulation. Edoxaban was initially withheld. The sudden onset of unilateral involuntary movements, together with the anatomically concordant left thalamic hemorrhage, supported a vascular cause. Diabetic striatopathy was considered less likely based on the overall biochemical and imaging findings. Admission capillary blood glucose was 6.5 mmol/L, and HbA1c was 52 mmol/mol. Subsequent MRI showed expected evolution of the left thalamic haematoma, together with chronic small-vessel cerebrovascular disease, multiple cerebral microhaemorrhages, and several small acute and subacute subcortical infarcts, without a reported imaging pattern suggestive of diabetic striatopathy [12]. Primary neurodegenerative, autoimmune, and medication-induced causes were also considered less likely because of the acute presentation and absence of relevant preceding features.

Tetrabenazine was started at 12.5 mg once daily and titrated during the admission because the involuntary movements were interfering with mobility and participation in rehabilitation. The maximum inpatient dose was 25 mg three times daily (75 mg/day), with good symptomatic improvement. The exact dates of the intermediate inpatient dose increases were not recoverable from the available records. An admission ECG on presentation showed sinus rhythm at 69 beats/min, with a QT interval of 367 ms and QTc of 393 ms. Medication review was documented during admission, and potential mood-related adverse effects were discussed with the patient at treatment initiation. No severe low mood, suicidal ideation, or parkinsonian adverse effects were documented during the inpatient stay. CYP2D6 genotyping was not documented in the available records. Repeat computed tomography approximately four weeks after the index scan showed a stable left thalamic hemorrhage without expansion or new intracranial hemorrhage.

Magnetic resonance imaging approximately six weeks after presentation showed expected evolution of the left thalamic hematoma on T2-weighted imaging (Figure 2), together with chronic small-vessel cerebrovascular disease, multiple cerebral microhemorrhages, and several small acute and subacute subcortical infarcts in the cerebral hemispheres.

**Figure 2:**
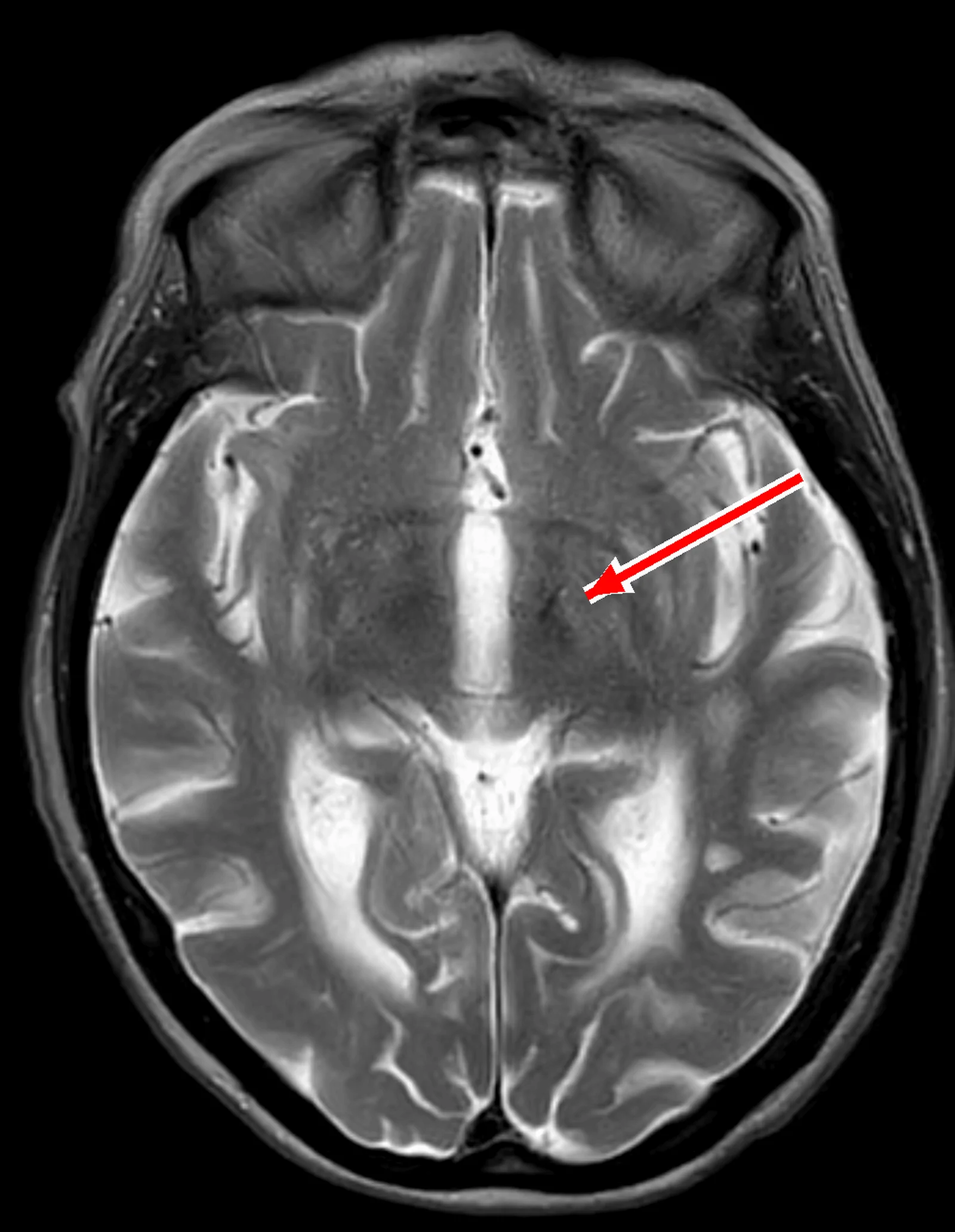
Evolution of the left thalamic hematoma on T2-weighted MRI. Axial T2-weighted MRI performed approximately six weeks after presentation demonstrating expected evolution of the left thalamic hematoma. The arrow indicates the site of the evolving left thalamic hematoma.

Susceptibility-weighted imaging demonstrated blooming at the site of the previous left thalamic hematoma, consistent with residual blood products (Figure 3). These additional abnormalities were identified on subsequent imaging after the initial presentation with hemichorea-hemiballismus. The deep thalamic location, longstanding hypertension, and concurrent edoxaban treatment favoured hypertensive small-vessel disease as the most likely cause of the haemorrhage, with anticoagulation contributing to the bleeding risk. The absence of skull fracture, extra-axial collection, or additional parenchymal haemorrhage on the initial CT made associated traumatic intracranial injury less likely. However, as no imaging was available immediately before or after the fall, a traumatic contribution or the possibility that the haemorrhage had already occurred and contributed to the fall or subsequent unsteadiness could not be completely excluded.

**Figure 3:**
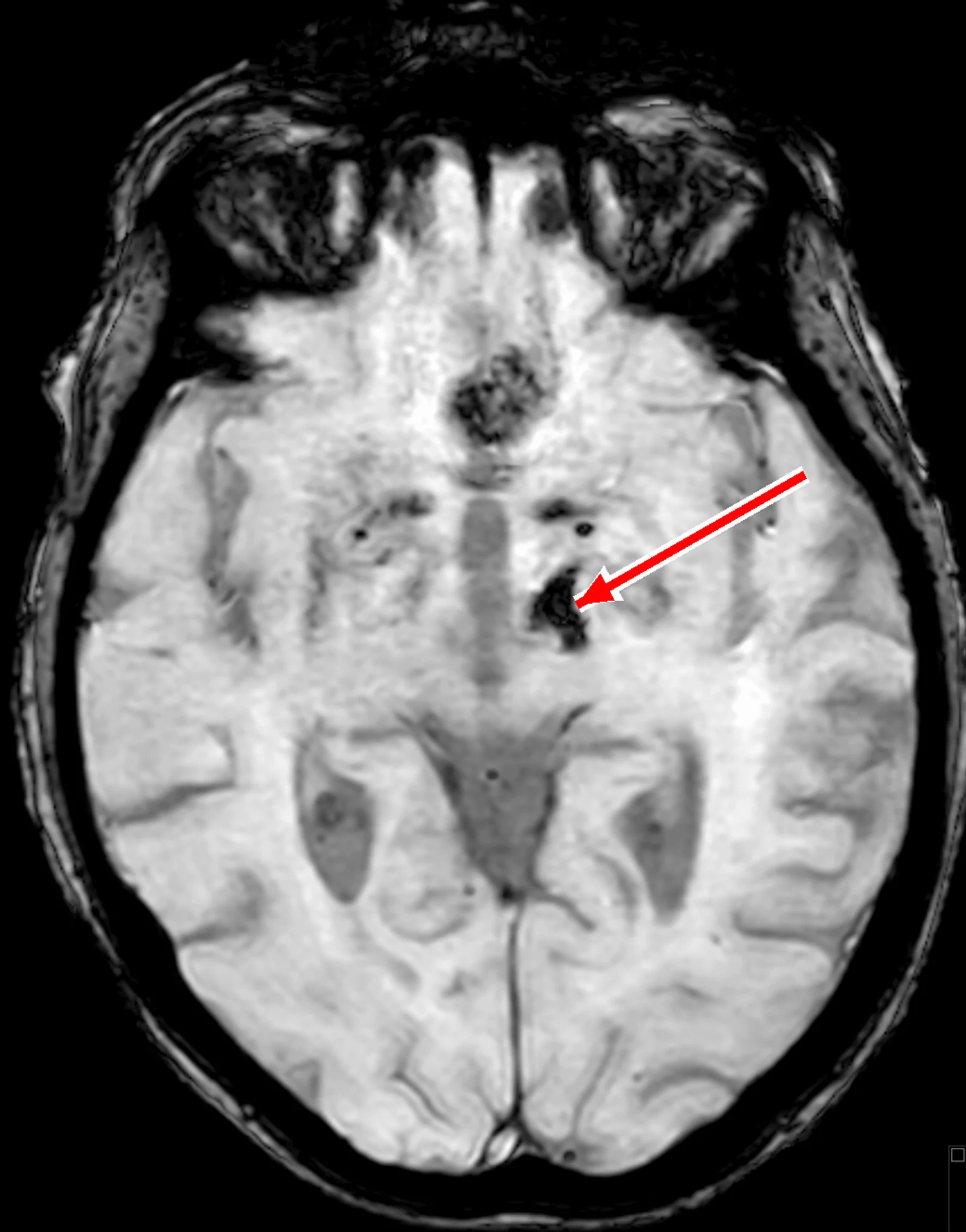
Susceptibility-weighted MRI of the left thalamic hematoma. Axial susceptibility-weighted MRI performed approximately six weeks after presentation demonstrating blooming at the site of the previous left thalamic hematoma, consistent with residual blood products. The arrow indicates the area of susceptibility blooming.

Following improvement during inpatient rehabilitation, tetrabenazine was gradually reduced to identify the lowest effective dose. An inadvertent dose reduction around discharge was followed by worsening of the hemichorea, and in late March 2025the dose was restored to 12.5 mg three times daily (37.5 mg/day), with good symptomatic control. This dose was maintained through early May. On May 2025**,** the morning dose was increased to 25 mg while the midday and evening doses remained 12.5 mg, giving a total dose of 50 mg/day. Following limited additional benefit, the midday dose was increased in late May, giving a regimen of 25 mg in the morning, 25 mg at midday, and 12.5 mg in the evening (62.5 mg/day). This was the final clearly documented stable tetrabenazine regimen and was still being taken at review on late June 2025. Because of increasing irritability, low mood, and emotional lability, a switch to aripiprazole was planned. Once aripiprazole had been established, tetrabenazine was tapered by 12.5 mg per week until withdrawal. By the October 2025 follow-up, tetrabenazine had been discontinued.

At early post-discharge review, while taking tetrabenazine 12.5 mg three times daily, the patient reported some agitation but denied significant low mood or suicidal thoughts. No formal standardized mood assessment was documented. By approximately five to six months after presentation, following further tetrabenazine dose escalation, the patient and her husband reported worsening irritability, low mood, and emotional lability, despite improved control of the chorea. These symptoms were considered at least partly related to tetrabenazine, and a change in treatment was agreed with the patient and her clinical team. Aripiprazole was subsequently introduced, and once established, tetrabenazine was gradually tapered until withdrawal.

At follow-up approximately nine months after the hemorrhage, the hemichorea was markedly improved while taking aripiprazole 7.5 mg daily, and her mood had improved after tetrabenazine was discontinued. The same dose was therefore continued. More than 12 months after the index event, mild right-sided involuntary movements remained clinically apparent and had increased slightly, so aripiprazole was increased to 10 mg daily. At this stage, she also reported persistent right-sided limb pain, which was considered compatible with possible central post-stroke pain of thalamic origin. Repeat neuroimaging was arranged because of concurrent gradual cognitive and gait decline.

Edoxaban had initially been withheld following the intracerebral hemorrhage, with a plan to avoid anticoagulation for at least one month. It was subsequently restarted 49 days after the index presentation after consideration of the competing thromboembolic risk associated with atrial flutter.

The longitudinal clinical course is summarized in Table 1.

**Table 1:** Longitudinal clinical and treatment course.

Time from index presentation	Clinical event
Presentation	Abrupt right foot choreiform/ballistic movements progressed to the right arm; no weakness, sensory loss, dysarthria, visual symptoms, headache, or impaired consciousness. CT showed a focal acute left thalamic hemorrhage. Four-factor prothrombin complex concentrate was administered for reversal of edoxaban-associated anticoagulation.
Early admission	Neurology confirmed right-sided hemichorea-hemiballismus. Tetrabenazine was started at 12.5 mg daily and titrated according to clinical response, reaching a maximum inpatient dose of 25 mg three times daily (75 mg/day). The exact duration of the intermediate inpatient doses was not available from the contemporaneous records.
Approximately 4 weeks	Repeat CT showed a stable left thalamic hemorrhage with no expansion or new intracranial hemorrhage.
Approximately 6 weeks	MRI showed expected evolution of the thalamic hematoma, chronic small-vessel disease, cerebral microhemorrhages, and several small acute/subacute subcortical infarcts.
Day 49	Edoxaban was restarted after consideration of the competing thromboembolic risk associated with atrial flutter.
Approximately 2-3 months	Late March–early May 2025: Hemichorea worsened following an inadvertent tetrabenazine dose reduction around discharge. The dose was restored to 12.5 mg three times daily (37.5 mg/day) on 28 March, with good symptomatic control, and this regimen remained documented through early May.
Approximately 4 months	May 2025: Because residual hemichorea continued to interfere with mobility and rehabilitation, tetrabenazine was increased on 19 May to 25 mg in the morning, 12.5 mg at midday, and 12.5 mg in the evening (50 mg/day). Following limited additional benefit, the dose was increased again in late May to a total of 62.5 mg/day (25 mg morning, 25 mg midday, and 12.5 mg evening).
Approximately 5-6 months	Late June 2025: Tetrabenazine 25 mg in the morning, 25 mg at midday, and 12.5 mg at night (62.5 mg/day) was confirmed as the current regimen. Although chorea was better controlled, the patient developed worsening irritability, low mood, and emotional lability. A switch to aripiprazole was planned.
Approximately 7 months	August 2025: Aripiprazole was started at 2.5 mg daily, with planned increases of 2.5 mg every two weeks to 7.5 mg daily. Once established on aripiprazole, tetrabenazine was tapered in 12.5-mg weekly reductions until discontinued.
Approximately 9 months	October 2025: Hemichorea was markedly improved on aripiprazole 7.5 mg daily, and mood had improved following discontinuation of tetrabenazine. Aripiprazole 7.5 mg daily was continued.
More than 12 months	Mild choreiform movements persisted and had increased slightly; aripiprazole was increased to 10 mg daily. Possible central post-stroke pain of thalamic origin was also considered.

## Discussion

Hemichorea-hemiballismus can result from disruption of a wider basal ganglia-thalamocortical motor network rather than injury to the subthalamic nucleus alone [5-7]. The thalamus is an important relay within this network, and a focal thalamic lesion may disturb connections between the basal ganglia and motor cortex, leading to contralateral hyperkinetic movements. This network-based explanation provides a plausible mechanism for the presentation in our patient [7,8].

In this patient, the sudden onset of right-sided choreiform and ballistic movements, together with the anatomically concordant left thalamic haemorrhage and the absence of another acute lesion on the initial CT, favoured the thalamic haemorrhage as the principal lesion associated with the movement disorder. Subsequent MRI demonstrated several small acute and subacute subcortical infarcts in the cerebral hemispheres; these were identified after the hemichorea-hemiballismus was already established, and their precise anatomical locations were not specified in the available radiology report. A contribution from these additional ischaemic lesions therefore cannot be completely excluded. The presentation was unusual because the movement disorder occurred without weakness, sensory loss, dysarthria, visual disturbance, or impaired consciousness. The imaging is best interpreted as showing a lesion centered within the thalamus rather than proving that all adjacent structures involved in the motor network were completely spared.

A recently published Cureus case described a 68-year-old man with hemiballismus following a left thalamic haemorrhagic stroke. Imaging showed a lesion centred within the thalamus with extension into the superior cerebral crus, and treatment with haloperidol, sodium valproate, and clonazepam resulted in only partial improvement during short-term follow-up [9]. The present case adds to this limited literature by documenting clinically observed, dose-related improvement with tetrabenazine, recurrence of symptoms after dose reduction, treatment-limiting neuropsychiatric adverse effects, subsequent treatment with aripiprazole, and persistence of hemichorea beyond 12 months. The prolonged follow-up and detailed treatment course are the main distinguishing features of this case.

Evidence for the treatment of post-stroke hemichorea-hemiballismus remains limited and is based mainly on case reports, small series, and experience from other hyperkinetic movement disorders [3,13]. Dopamine receptor-blocking agents and vesicular monoamine transporter 2 inhibition with tetrabenazine are recognized symptomatic treatments for severe chorea and ballism [13]. Tetrabenazine has also been reported to improve post-stroke choreiform movement disorders, including after thalamic stroke [14]. Treatment response was assessed clinically and through patient and carer reports; serial standardised movement, mood, or functional outcome scores and standardised video assessments were not available. Nevertheless, improvement following dose escalation, worsening after dose reduction, and improvement when treatment was restored supported a clinically observed, dose-related treatment effect. However, this benefit was eventually limited by significant mood and behavioral adverse effects.

Neuropsychiatric adverse effects are well recognized with tetrabenazine. In a large retrospective series of patients receiving tetrabenazine for hyperkinetic movement disorders, depression was among the reported adverse effects [15]. In this patient, irritability, low mood, and emotional lability became more prominent during higher-dose treatment and improved after tetrabenazine was withdrawn. Although a direct causal relationship cannot be established, the timing made a medication-related contribution likely. This underlines the importance of considering mood and behavior, as well as control of involuntary movements, when assessing treatment response.

Aripiprazole is not an established treatment for post-stroke hemichorea, and the response in this case should not be interpreted as evidence that it is superior to tetrabenazine. However, a small comparative study in Huntington disease reported improvement in chorea with both aripiprazole and tetrabenazine, with better tolerability in the aripiprazole group [16]. In our patient, switching to aripiprazole was associated with continued improvement in hemichorea, while her mood improved after tetrabenazine was stopped. As treatment response was not assessed using standardised movement scores and post-stroke chorea may improve naturally over time, the degree to which aripiprazole contributed to the observed improvement remains uncertain. Although the evidence for aripiprazole in post-stroke hemichorea remains very limited, it may be considered as an alternative when tetrabenazine is effective but poorly tolerated.

The cause of the intracerebral hemorrhage also warrants consideration. The patient had longstanding hypertension and was taking edoxaban at the time of presentation. The deep thalamic hemorrhage was considered most likely related to hypertension, with concurrent edoxaban treatment contributing to the risk of bleeding. Magnetic resonance imaging later demonstrated cerebral microhemorrhages, chronic small-vessel disease, and several small acute and subacute ischemic lesions. Anticoagulation was subsequently restarted after multidisciplinary assessment because of the competing thromboembolic risk. Although the later MRI findings demonstrated more widespread cerebrovascular disease, the hemichorea-hemiballismus was already fully established before these additional abnormalities were identified. The timing and anatomical concordance of the thalamic haemorrhage therefore favoured it as the main lesion associated with the initial movement disorder, although a contribution from the additional cerebrovascular lesions cannot be excluded.

From a practical perspective, sudden unilateral chorea or ballism should prompt urgent neuroimaging even when strength, sensation, speech, and consciousness are preserved. When a vascular cause is identified, treatment should be guided by the extent to which the involuntary movements interfere with mobility, rehabilitation, and daily activities, while also taking medication adverse effects into account. Continued follow-up is important when symptoms persist, as both the severity of the movement disorder and the balance between treatment benefit and adverse effects may change over time.

## Conclusions

Acute thalamic intracerebral hemorrhage can present with isolated hemichorea-hemiballismus in the absence of weakness, sensory loss, or other typical focal neurological deficits. In this patient, the movement disorder remained clinically relevant for more than 12 months. Tetrabenazine produced substantial symptomatic improvement, with worsening when the dose was reduced, but its continued use was limited by prominent mood and behavioral adverse effects. After tetrabenazine was withdrawn, the involuntary movements remained clinically improved during treatment with aripiprazole, while her mood also improved. However, in the absence of standardised movement scores and given the possibility of natural recovery over time, the contribution of aripiprazole to this improvement cannot be established with certainty. This case emphasizes the importance of urgent neuroimaging in patients presenting with sudden unilateral hyperkinetic movements and the need for continued, individualized management when post-stroke hemichorea persists.
